# Feeding Difficulties and Gastrostomy in Dravet Syndrome: A UK-Wide Survey and 2-Center Experience

**DOI:** 10.1212/CPJ.0000000000200288

**Published:** 2024-04-23

**Authors:** Lisa M. Clayton, Bahar Azadi, Claire Eldred, Galia Wilson, Robert Robinson, Sanjay M. Sisodiya

**Affiliations:** UCL Queen Square Institute of Neurology (LMC, SMS), London; Chalfont Centre for Epilepsy (LMC, SMS), Bucks; Great Ormond Street Hospital (BA, RR), London; and Dravet Syndrome UK (CE, GW), Registered Charity Number 1128289, Member of Dravet Syndrome European Federation, Chesterfield, United Kingdom.

## Abstract

**Background and Objectives:**

Dravet syndrome (DS) is one of the most common monogenic epilepsies. Alongside the core seizure and developmental phenotypes, problems with appetite, swallowing, and weight loss are frequently reported, necessitating gastrostomy in some. We explored the burden of feeding difficulties and need for gastrostomy across 3 DS populations in the United Kingdom. We document caregiver opinion and postgastrostomy outcomes, and provide guidance regarding feeding issues and gastrostomy in DS.

**Methods:**

A retrospective, observational study was conducted; data were collected from medical records of 124 individuals with DS attending clinics at the National Hospital for Neurology and Neurosurgery, and Great Ormond Street Hospital, and from 65 DS caregiver responses to a UK-wide survey.

**Results:**

In total, 64 of 124 (52%) had at least 1 feeding difficulty; 21 of 124 (17%) had a gastrostomy, and gastrostomy was being considered in 5%; the most common reasons for gastrostomy were poor appetite (81%) and weight loss/failure to gain weight (71%). Median age at gastrostomy was 17 years (range 2.5–59). Multivariate analyses identified several factors that in combination contributed to risk of feeding difficulties and gastrostomy, including treatment with several antiseizure medications (ASMs), of which stiripentol made a unique contribution to risk of gastrostomy (*p* = 0.048, odds ratio 3.20, 95% CI 1.01–10.16). Preinsertion, 88% of caregivers were worried about the gastrostomy, with concerns across a range of issues. Postgastrostomy, 88% of caregivers were happy that their child had the gastrostomy, and >90% agreed that the gastrostomy ensured medication compliance, that their child's overall health was better, and that quality of life improved.

**Discussion:**

Feeding difficulties are common in DS, and 17% require a gastrostomy to address these. Risk factors for feeding difficulties in DS are unknown, but ASMs may play a role. There is a high level of caregiver concern regarding gastrostomy preprocedure; however, postgastrostomy caregiver opinion is positive. Feeding difficulties should be proactively sought during review of people with DS, and the potential need for gastrostomy should be discussed.

## Introduction

Dravet syndrome (DS) is a developmental and epileptic encephalopathy (DEE), associated with variants in the voltage-gated sodium channel alpha 1 subunit (*SCN1A*) gene. It is one of the most common monogenic epilepsies, with an incidence of 1 of 15,500.^1^

Alongside the core seizure and developmental phenotypes of DS, problems with appetite, swallowing, and weight loss are frequently reported^2-7^ (eTable 1). In one of the largest surveys of comorbidities in DS, 99% of caregivers reported at least 1 concern relating to feeding, making it the most frequently reported category of concern.^2^ In a population-based study in Norway, 25% of individuals with DS were reported to have a “gastrotube”.^8^ Similarly, in a survey of comorbidities in DEEs, 12% of respondents reported that their child with DS had a gastrostomy.^9^ In a series describing 22 adults with DS, 18% had a gastrostomy.^10^

Despite their frequency and a negative effect on quality of life, problems with appetite, swallowing, and weight loss (referred to collectively as “feeding difficulties”) have received little attention in DS, and the reasons, and potential risk factors, for gastrostomy are unknown.

In this retrospective, observational study, we explored the burden of feeding difficulties and the need for gastrostomy across 3 DS populations in the United Kingdom. We document caregiver opinions and concerns regarding gastrostomy, and provide guidance regarding feeding issues and gastrostomy for those involved in the care of individuals with DS.

## Methods

### Standard Protocol Approvals, Registrations, and Patient Consents

This work formed part of a service evaluation registered and independently approved by the Clinical Audit and Quality Improvement Subcommittee, Queen Square Division, University College London Hospitals NHS Trust, and the Audit and Quality Improvement Subcommittee at the Great Ormond Street Hospital (GOSH) NHS Trust. These approvals waive the need for approval by an ethics committee, in accordance with UK legislation and NHS operating procedures.

### Study Design and DS Cohorts

A retrospective, observational study was conducted. Data were collected from 3 DS populations: adults attending clinics at the National Hospital for Neurology and Neurosurgery (“adult DS cohort”), children attending clinics at GOSH (“pediatric DS cohort”) (both tertiary epilepsy services), together forming the “combined DS cohort”, and DS caregivers responding to a UK-wide survey conducted by Dravet Syndrome UK (DSUK), an independent, patient advocacy group (Charity Number 1128289) (“survey cohort”). The “pediatric DS cohort” and “adult DS cohort” were combined so as to gain an understanding of the burden of feeding difficulties and the need for gastrostomy across a DS population as a whole (regardless of age).

Individuals in the combined DS cohort were identified from hospital records and departmental databases of individuals with DS. All clinical and genetic information was extracted from the medical records. Individuals with a clinical diagnosis of DS due to a causal *SCN1A* variant were included. “Causal *SCN1A* variants” were those that had been determined as disease-causing by the diagnostic genetic laboratory of the submitting center, including those classified as disease-causing by the submitting center before the development of American College of Medical Genetics criteria.^11^ Individuals with DS due to a causal variant in an alternative gene (i.e., not *SCN1A*), or those in whom genetic testing had not revealed a causal variant, were excluded. For the survey cohort, all information was obtained from the survey responses. Caregivers were asked to state whether their child was known to have an *SCN1A* variant.

Children/childhood was defined as those aged younger than 18 years.

### DSUK Survey

The anonymous, cross-sectional survey regarding feeding difficulties and gastrostomy was developed by adult neurologists working with individuals with DS (LMC, SMS), alongside representatives, and caregivers, from DSUK (CE, GW). The survey was circulated through email and private Facebook group to DSUK-registered families, and open for responses between 7th and 28th January 2022. Survey questions were multiple choice, including Likert scale responses, with opportunities for open-ended responses (Supplementary Material).

### Defining Feeding Difficulties

The term “feeding difficulty” was used to define any symptom that could reflect difficulties in the normal process of providing adequate food, drink, and medication to an individual, regardless of etiology, severity, or consequences.^12^ The term “feeding difficulty/problem” has been suggested as the preferred term to use when referring to eating behaviors that are unrelated to body image concerns yet may impair functioning.^13^

Weight loss and failure to gain weight were included as feeding difficulties because these can reflect the consequences of inadequate nutritional intake. Medication refusal was included because this can pose a significant challenge in individuals with drug-resistant epilepsy and intellectual disability, at times necessitating gastrostomy,^14^ and in our experience, frequently occurs alongside “poor appetite” in people with DS.^15^ Sialorrhea was included because this can reflect oropharyngeal dysfunction.

### Defining Postgastrostomy Outcomes

For the combined DS cohort, a “positive overall outcome” was ascribed when it was documented in the medical records that there had been improvements in aspects of quality of life, health, nutrition, medication compliance, weight, and/or seizure control.

### Data Analysis

Data from the survey cohort were analyzed separately throughout due to differences in data acquisition methods. Caregivers of individuals included in the combined DS cohort may have completed the anonymized DSUK survey, and therefore, some individuals may be included in both the survey cohort and combined DS cohort, a further reason to analyze the cohorts separately.

“Current” antiseizure medication (ASM) refers to ASM taken at the time of last follow-up/survey in those without gastrostomy, and ASMs taken at the time of gastrostomy insertion in those with gastrostomy. For statistical analyses, only ASMs taken by ≥10% of either cohort were included.

Data were analyzed using IBM SPSS Statistics for Windows. Mean and SD are given where data are normally distributed, and median and interquartile ranges (IQR) otherwise. The appropriate parametric or nonparametric tests were used to compare data between groups. The Bonferroni-Dunn method of correction was used for multiple comparisons, with significance set at *p* ≤ 0.05. Binary logistic regression was used to investigate groups of factors that might be associated with feeding difficulties and gastrostomy. A multivariate model was built using univariate associations with a *p* < 0.25.^16^ Variables showing multicollinearity (variance inflation factor ≥5) were excluded from the model.

### Data Availability

Anonymized data not published within this article will be made available by request from any qualified investigator.

## Results

### Demographics

#### Combined DS Cohort

One hundred forty individuals with *SCN1A*-related DS were identified. One adult was excluded because of a history of dysphagia secondary to nasopharyngeal carcinoma. Fifteen children were excluded because of a lack of available clinical information. One hundred twenty-four individuals were included. Demographic and clinical details are provided in Table 1. Feeding difficulties in 1 individual have been reported previously.^15^

**Table 1:** Demographic and Clinical Details of the DS Cohorts

	Adult DS cohort (N = 52)	Pediatric DS cohort (N = 72)	Combined DS cohort (N = 124)	Survey cohort (N = 65)
Female sex, n (%)	30 (58)	37 (51)	67 (54)	26 (40)
Median age at last follow-up/time of survey (y)	28.5	7.0	16.0	15.0
	Range: 18–70	Range: 1–20	Range: 1–70	Range: 3–42
	IQR: 22.3–39	IQR: 5–12	IQR: 6–25	IQR: 8–22
Children (<18), n (%)	0	68 (94)	68 (55)	38 (58)
Diagnosis of ASD, n (%)	20 (38)	12 (17)	32 (26)	31 (48)^a^
*SCN1A* variant type, n (%)				65/65 reported to have an *SCN1A* variant, but variant details were not obtained from the survey
Missense	22 (42)	28 (39)	50 (40)	
Stopgain	11 (21)	14 (19)	25 (20)	
Splice site	9 (17)	8 (11)	17 (14)	
Frameshift deletion	6 (12)	5 (7)	11 (9)	
Frameshift insertion	2 (4)	1 (1)	3 (2)	
Inframe indel	1 (2)	4 (6)	5 (4)	
Whole-gene deletion	1 (2)	0	1 (1)	
Unknown	0	12 (20)	12 (10)	
Null variants, n (%)^b^	20 (38)	20 (28)	40 (32)	
Median number of ASMs currently taking	3	3^c^	3^c^	3
	Range: 1–5	Range: 0–5	Range: 0–5	Range: 1–5
	IQR: 3–4	IQR: 2–4	IQR: 2.3–4	IQR: 2–3.5
ASMs currently taking, n (%)				
Valproate	41 (79)	50 (69)	91 (73)	45 (69)
Clobazam	28 (54)	39 (54)	67 (54)	49 (75)
Stiripentol	17 (33)	41 (57)	58 (47)	40 (62)
Cannabidiol	7 (13)	16 (22)	23 (19)	10 (15)
Fenfluramine^d^	0	12 (17)	12 (10)	5 (7)
Levetiracetam	21 (40)	9 (13)	30 (24)	10 (15)
Topiramate	13 (25)	17 (24)	30 (24)	13 (20)
Ketogenic diet	2 (4)	5 (7)	7 (6)	4 (6)
Zonisamide	8 (15)	0	8 (6)	3 (5)
Perampanel	4 (8)	1 (1)	5 (4)	1 (2)
Clonazepam	3 (6)	3 (4)	6 (5)	4 (6)
Brivaracetam	3 (6)	0	3 (2)	2 (3)
Potassium bromide	0	3 (4)	3 (2)	1 (2)
Lacosamide	6 (12)	1 (1)	7 (6)	0
Carbamazepine/oxcarbazepine	4 (8)	0	4 (3)	0
Lamotrigine	3 (6)	0	3 (2)	2 (3)
Other	3 (6)^e^	3 (4)^f^	6 (5)	3 (5)^g^
Unknown	0	4 (6)	4 (3)	0

Abbreviations: ASD = autism spectrum disorder; ASMs = antiseizure medications; DS = Dravet syndrome; IQR = interquartile range; n = number of individuals; unknown = information not available in medical records; y = year(s).

a Information not available for 6 individuals.

b Includes gene deletions, stopgains, frameshift deletions, and frameshift insertions.

c Information not available for 4 individuals.

d At the time of this study, fenfluramine had not been recommended for use in the United Kingdom by the National Institute for Health and Care Excellence for the treatment of seizures in DS, and children receiving fenfluramine were doing so through compassionate use, early access programmes, or clinical trials.

e Piracetam (1 individual), primidone (2 individuals).

f Phenobarbital (3 individuals).

g Phenobarbital (2 individuals), rufinamide (1 individual).

#### Survey Cohort

Seventy-three caregivers responded to the survey. Eight were excluded (5 with incomplete data, 1 without a confirmed DS diagnosis, and 2 without *SCN1A* variants). Sixty-five responses were included. Demographic and clinical details are provided in Table 1.

### Feeding Difficulties

#### Combined DS Cohort

Sixty-four of 124 had at least 1 feeding difficulty, with a median age at onset of 13 years. Poor appetite, weight loss, and failure to gain weight were the most frequently reported symptoms. Medication refusal was reported in 16 of whom 12 (75%) also reported poor appetite (Table 2).

**Table 2:** Feeding Difficulties and Gastrostomy Across the DS Cohorts

	Adult DS cohort (N = 52)	Pediatric DS cohort (N = 72)	Combined DS cohort (N = 124)	Survey cohort (N = 65)
Feeding difficulty, n (%)				
Yes	31 (60)	33 (46)	64 (52)	60 (92)
No	21 (40)	38 (53)	59 (47)	5 (8)
Unknown	0	1 (1)	1 (1)	0
Type of feeding difficulty reported, n (%)				
Fussy/picky eater	2 (4)	12 (17)	14 (11)	42 (65)
Poor appetite	23 (44)	17 (24)	40 (32)	41 (63)
Weight loss	20 (38)	16 (22)	36 (29)	32 (49)
Being underweight/failure to gain weight	0	22 (31)	22 (18)	24 (37)
Swallowing difficulties	8 (15)	15 (21)	23 (19)	32 (49)^c^
Refusing medication	8 (15)	8 (11)	16 (13)	27 (42)
Chewing difficulties	3 (10)	1 (1)	4 (3)	29 (45)
Other	3 (6)^a^	2 (3)^b^	3 (2)	See eFigure 1
Median age at onset of feeding difficulties (y)	25.5	4	13	3
	Range: 7–58	Range: 0.5–16	Range: 0.5–58	Range: 0–31
	IQR: 19.3–36	IQR: 2–8	IQR: 3–25	IQR: 2–7
Developed feeding difficulties in childhood, n (%)	3/31 (10)	33/33 (100)	36/64 (56)	55/60 (92)
Age at onset of feeding difficulties unknown, n	3	2	5	1
Gastrostomy, n (%)				
Yes	11 (21)	10 (14)	21 (17)	26 (40)
No	39 (75)	58 (80)	97 (78)	39 (60)
Being considered	2 (4)	4 (6)	6 (5)	0
Regarding individuals with gastrostomy	**N = 11**	**N = 10** ^f^	**N = 21** ^f^	**N = 26**
Median age at gastrostomy insertion (y)	22	3.5	17	7
	Range: 17–59	Range: 2.5–11	Range: 2.5–59	Range: 2–33
	IQR: 18–46	IQR: 2.9–5.3	IQR: 3.5–27	IQR: 4–11.3
Gastrostomy inserted in childhood, n (%)	1 (9)	10 (100)	11 (52)	22 (85)
Feeding symptoms leading to the need for gastrostomy^e^, n (%)				
Poor appetite/refusing food and/or drink	11 (100)	6 (60)^g^	17 (81)	22 (85)
Weight loss/failure to gain weight	10 (91)	5 (50)^g^	15 (71)	18 (69)
Difficulty swallowing	3 (27)	4 (40)^h^	7 (33)	8 (31)
Refusing medication	2 (18)	2 (20)	4 (19)	14 (54)
Aspiration pneumonia	2 (18)	1 (10)	3 (14)	7 (27)
Increased seizures due to missed medication	0	0	0	6 (23)
Other feeding symptoms	0	3 (30)^i^	3 (14)	1 (3.8)^j^
Median time between feeding symptom(s) onset and gastrostomy insertion (y)	1^d^	1	1	1.5
	Range: 1–10	Range: 0–4.5	Range: 0–10	Range: 0–31
	IQR: 1–5	IQR: 0.5–1.3	IQR: 1–3.5	IQR: 0.4–6.9
Periprocedural complications, n (%)				
Yes	3 (27)	0	3 (14)^k^	6 (23)^m^
No	7 (64)	10 (100)	17 (81)	20 (77)
Unknown	1 (9)	0	1 (5)	0
Long-term complications, n (%)				
Yes	4 (36)	3 (30)	7 (33)^l^	19 (73)^n^
No	6 (55)	7 (70)	13 (62)	7 (27)
Unknown	1 (9)	0	1 (5)	0
Mean time between gastrostomy insertion and last follow-up/survey (y)	3.4	3.8		6.1
	SD ± 3.4	SD ± 3.1		SD ± 3.6
	Range: 0–11	Range: 0–9		Range: 0.5–15

Abbreviations: DS = Dravet syndrome; IQR = interquartile range; n = number of individuals; y = year(s).

a Nausea (2 individuals), slow eating (1 individual).

b Nausea and vomiting (1 individual), difficulty tolerating ketogenic diet (1 individual).

c Includes “difficulty swallowing food” and/or “difficulty swallowing drink”.

d Rounded to the nearest year, as unable to determine more precise timelines.

e 18 individuals from the combined DS cohort and 24 from the survey cohort reported >1 feeding symptom that led to the need for gastrostomy.

f Feeding symptoms that led to gastrostomy were unknown for 1 individual.

g Includes 1 individual whose symptoms developed following status epilepticus.

h Includes 2 individuals whose symptoms developed following status epilepticus.

i “A decline in overall health” (1 individual) to facilitate treatment with the ketogenic diet (2 individuals: of note, poor appetite in both, and weight loss in 1, also contributed to the need for gastrostomy).

j “To manage ketogenic diet” (of note, “poor appetite/refusing food and/or drink” also contributed to the need for gastrostomy).

k Periprocedural complications included difficulties tolerating gastrostomy feeding, bleeding at the gastrostomy site, and peristomal skin infection (all in 1 individual), the need for “further manipulation of the tube” (1 individual), and pneumonia requiring treatment with intravenous antibiotics (1 individual).

l Six individuals had a peristomal skin infection requiring antibiotics, one of whom also caused nonaccidental damage to the external gastrostomy tube requiring insertion of a new tube. One individual developed peristomal pain and leaking, necessitating gastrostomy removal; in this individual, feeding symptoms have continued and are being monitored.

m Three had infections, one of whom required a “10-day hospital stay”. One individual had “difficulties with gastrostomy placement due to atypical anatomy”, and 1 had difficulty tolerating feed at the required rate. No details were provided for the remaining individual.

n Peristomal skin infection requiring antibiotics (17 individuals), granulation tissue (6), external tube splitting (3), peristomal pain (2), tube blocking (1), perisomal leaking (1), and “tube too tight” (1). In 3 individuals, details were not provided.

Fifteen of 64 individuals required a hospital admission because of feeding difficulties of whom 8 (53%) went on to have a gastrostomy and 3 (20%) are currently being considered for gastrostomy (eTable 2). Only 38 of 64 had been reviewed by a speech and language therapist (SALT) and 44 of 64 by a dietician. Twenty-four of 64 had undergone investigations or specialist review regarding the cause of their feeding difficulties (eTable 3). One individual with poor appetite and vomiting was identified to have valproate-induced hyperammonemia, with symptoms resolving after ASM changes. No additional medical cause of feeding difficulties (other than dysphagia) was identified in any individual.

For 13 of 20 individuals in the adult DS cohort who reported weight loss, information regarding the degree of weight loss was available. The median percentage body weight lost (from baseline weight to the lowest recorded weight) was 18% (range 9%–38%); 12 individuals (92%) experienced >10% weight loss. The mean body mass index (BMI) taken at the lowest weight was 17.8 (SD ± 3.6, range 13–25). In 9, BMI was below the normal range for adults (18.5–24.9).

#### Survey Cohort

Sixty of 65 caregivers reported at least 1 feeding difficulty, with individuals experiencing a median of 8 different feeding symptoms (range 1–16). The most common symptoms were “fussy/picky eater” and “poor appetite” (Table 2; eFigure 1). Twenty-seven of 65 individuals reported “often refusing medication” all of whom also reported “poor appetite”, “often refusing food”, and/or “often refusing drink”.

Thirty-nine of 60 individuals with feeding difficulties had been reviewed by SALT, and 21 of 60 had undergone medical investigations into the cause of their feeding difficulties (eTable 3).

### Gastrostomy

#### Combined DS Cohort

Seventeen percentage of individuals had a gastrostomy, and gastrostomy was being considered in a further 5%. The median age at insertion was 17 years. Poor appetite, and weight loss and/or failure to gain weight were the symptoms that most frequently led to the need for gastrostomy (Table 2).

#### Survey Cohort

Forty percentage of respondents reported that their child had a gastrostomy. “Poor appetite/refusing food and/or drink” and “weight loss/failure to gain weight” were the symptoms that most frequently led to the need for gastrostomy (Table 2).

### Risk Factors for Feeding Difficulties and Gastrostomy

#### Combined DS Cohort

After correction for multiple comparisons, no studied factors were found to be individually associated with the presence of feeding difficulties or the need for gastrostomy (eTable 4).

Binary logistic regression was performed to identify groups of factors that potentially associated with feeding difficulties and gastrostomy (eTable 4). For factors predicting the presence of feeding difficulties, the full model containing age, number of ASMs, sex, cannabidiol, clobazam, and topiramate treatment was statistically significant (χ^2^ (6, N = 120) = 20.14, *p* = 0.003) and correctly predicted 68% of cases. Only age made a unique contribution (*p* = 0.026, odds ratio (OR) 1.03, 95% CI 1.01–1.06). For factors predicting the need for gastrostomy, the full model containing number of ASMs, clobazam, and stiripentol treatment was statistically significant (χ^2^ (3, N = 120) = 10.08, *p* = 0.018) and correctly predicted 83% of cases. However, only treatment with stiripentol made a unique contribution (*p* = 0.048, OR 3.20, 95% CI 1.01–10.16).

#### Adult DS Cohort

In 16 of 31 individuals with feeding difficulties, the introduction of a new medication, or an increase in the dose of an existing medication, coincided with the onset of feeding difficulties. ASMs were implicated in 14 (topiramate, zonisamide, valproate, stiripentol, cannabidiol, and phenobarbitone) and sertraline in 2 individuals. In 13 of 16, implicated medications were reduced or withdrawn by the physician in an attempt to improve feeding difficulties, resulting in a resolution of symptoms in 5 of 13 (38%). In 7 of 13 (56%), symptoms continued despite medication changes, with 3 individuals going on to require gastrostomy. In 1 individual, no information about the effect of medication changes was available.

#### Pediatric DS Cohort

Three individuals required a gastrostomy due to feeding difficulties that developed following status epilepticus; 1 individual sustained a hypoxic-ischemic brain injury and developed multiple feeding difficulties, requiring a gastrostomy due to dysphagia; 1 individual developed dysphagia following an intensive care admission; and in 1 individual, there was developmental regression with poor appetite and weight loss following status epilepticus.

#### Survey Cohort

Only 5 individuals in the survey cohort reported no feeding difficulties, so statistical analysis was not undertaken. After correction for multiple comparisons, valproate treatment was the only factor associated with the need for gastrostomy (adjusted *p* = 0.01) (eTable 4).

Binary logistic regression was performed to explore factors predicting the need for gastrostomy. The full model containing number of ASMs and stiripentol treatment was statistically significant (χ^2^ (2, N = 63) = 8.44, *p* = 0.015) and correctly predicted 64% of cases. No individual variable made a unique contribution.

In 18 individuals with feeding difficulties, ASMs (clobazam, topiramate, valproate, stiripentol, zonisamide, and fenfluramine) were reduced or withdrawn in an attempt to improve feeding symptoms, resulting in an improvement in symptoms in 12. Nine of 18 (50%) still went on to require a gastrostomy, including 4 who reported some improvement in symptoms after the medication changes.

A wide range of feeding symptoms were reported in those with, and without, gastrostomy. After correction for multiple comparisons, the symptom “often refusing medication” was the only symptom associated with the need for gastrostomy (χ^2^ test, adjusted *p* = 0.03, OR 6.25, 95% CI 2.02–19.32) (eTable 5).

### Postgastrostomy Complications and Outcomes

#### Combined DS Cohort

Periprocedural complications (i.e., those occurring during, or in the days following, the procedure) occurred in 14% (Table 2). Long-term complications were reported in 33% (Table 2).

In 18 of 21, gastrostomy insertion led to a positive overall outcome. In 11 of 21, there was an improvement in seizure control. Fifteen of 18 individuals in whom oral intake is permitted still take a proportion of their food/fluid orally postgastrostomy (eTable 6).

#### Survey Cohort

Periprocedural complications were reported in 23%. Long-term complications were reported in 73% (Table 2).

**Figure 1:**
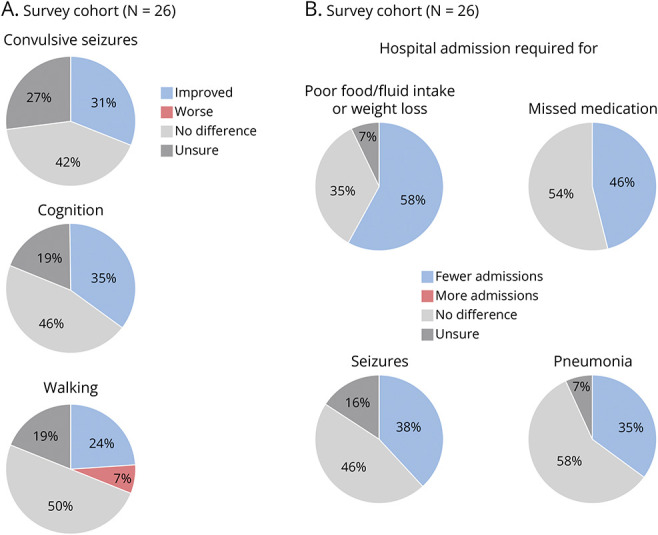
Postgastrostomy Outcomes in the Survey Cohort (A) Caregivers were asked if convulsive seizures, cognition, and walking were improved, worse, or no difference postgastrostomy. (B) Caregivers were asked if postgastrostomy, there was a change in the number of hospital admissions required for seizures, missed medication, pneumonia, and poor food/fluid intake and/or weight loss.

Caregivers reported positive postgastrostomy outcomes across several domains including those relating to seizure control, cognition, and hospital admissions (Figure 1).

### Caregiver Opinion Regarding Gastrostomy (Survey Cohort)

#### Caregiver Opinion Pregastrostomy

**Figure 2:**
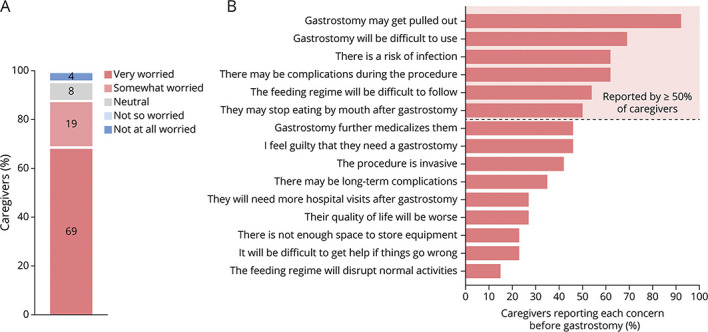
Caregiver Concerns Pregastrostomy Caregivers from the survey cohort were asked about their level of concern regarding gastrostomy before the procedure (A) and to indicate if they had specific concerns, across of range of issues, relating to gastrostomy (B).

Eighty-eight percentage of caregivers reported that they were worried about the gastrostomy before the procedure, with high levels of concern expressed across a wide range of issues (Figure 2).

Fourteen of 26 caregivers reported that they had no access to information regarding gastrostomy. Twenty-one of 26 reported that they would have liked access to additional information, including 20 of 26 who would have liked access to information specifically relating to gastrostomy in DS.

#### Caregiver Opinion Postgastrostomy

**Figure 3:**
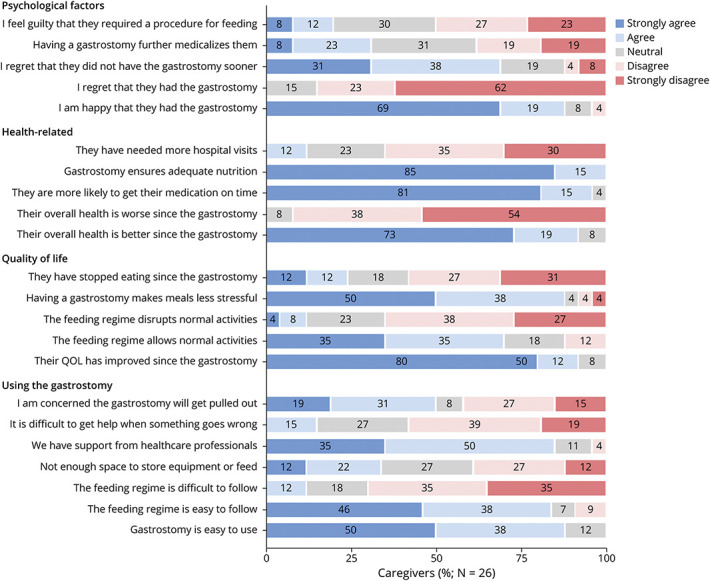
Caregiver Opinion Postgastrostomy Caregivers from the survey cohort were asked whether they strongly agreed (dark blue), agreed (light blue), neither agreed or disagreed/neutral (gray), disagreed (pink), or strongly disagreed (red), with several statements regarding life for them and their child with DS postgastrostomy.

**Figure 4:**
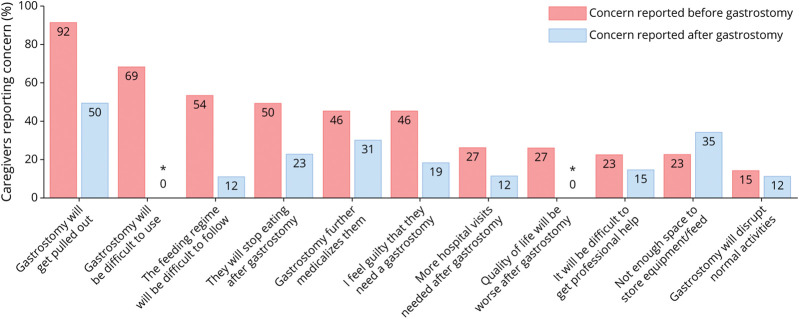
Caregiver Concerns Pregastrostomy and Postgastrostomy Comparison of caregiver concerns from the survey cohort (N = 26) pregastrostomy (red) and postgastrostomy (blue). * = pregastrostomy, the statement given to caregivers was “the gastrostomy will be difficult to use”; postgastrostomy, the statement given was “the gastrostomy is easy to use”. In the figure, the percentage of caregivers who *disagreed* or *strongly disagreed* with the statement “the gastrostomy is easy to use” was used for the postgastrostomy comparison. # = pregastrostomy, the statement given to caregivers was “quality of life will be worse with a gastrostomy”; postgastrostomy, the statement given was “quality of life has improved since the gastrostomy”. In the figure, the percentage of caregivers who *disagreed* or *strongly disagreed* with the statement “quality of life has improved since the gastrostomy” was used for the postgastrostomy comparison.

Caregiver opinion postgastrostomy was positive across areas relating to quality of life, health, and practical aspects of using the gastrostomy (Figure 3). When comparing concerns pregastrostomy and postgastrostomy, there were fewer concerns across almost all areas postgastrostomy (Figure 4).

Fifteen of 26 caregivers provided comments regarding their opinions and experience of gastrostomy, the majority of which were positive. Negative comments related to a lack of professional support and awareness of feeding issues in DS (eTable 7).

## Discussion

We show that feeding difficulties are common in DS and that individuals often experience multiple symptoms across a wide range of feeding issues. Gastrostomy is frequently required; across a population of children and adults with DS in the United Kingdom, 17% had a gastrostomy and gastrostomy was being considered in a further 5%. These reflect rates recorded in a registry-based population study of 53 individuals with DS in Norway, where 25% had a “gastrotube”.^8^

The occurrence of feeding difficulties in DS (eTable 1) and that gastrostomy may be required^8-10^ has been reported previously, but these studies were intended to explore comorbidities in general, or other aspects of DS, and none have specifically investigated feeding difficulties in DS. No studies have explored risk factors for, or the consequences of, feeding difficulties in DS, the reasons for gastrostomy insertion, or postgastrostomy outcomes in DS. Most DS caregiver surveys reporting feeding difficulties have been limited to children^2,3,5,17^ (a common shortfall in DS research),^18^ and none have included “older” adults (≥40 years) (eTable 1). This study includes both children and adults with DS, showing that feeding difficulties (and the need for gastrostomy insertion) can emerge at any age, from early infancy to late adulthood.

The underlying causes of feeding difficulties and risk factors for the need for gastrostomy in DS are unknown, and likely to be multifactorial.^15^ We identified several factors that in combination contributed to the risk of feeding difficulties and gastrostomy, including treatment with various ASMs. This is not surprising; some of the most frequently used therapies in DS, including topiramate,^19^ stiripentol,^20^ cannabidiol,^21^ ketogenic diet,^22^ and fenfluramine,^23^ are all associated with decreased appetite and weight loss, and treatment with stiripentol has previously been associated with poor appetite in DS.^6^ We found that in some individuals, medication changes coincided with the onset of feeding difficulties. We are unable to determine whether the feeding difficulties developed because of the medication change, or whether they were part of an underlying problem, worsening seizure control, or overall deterioration that led to the decision to undertaken medication changes. However, despite implicated medications being reduced/withdrawn, 23–50% still went on to require gastrostomy. Overall, these data suggest that although ASM changes may be implicated in, or be markers for, the development of feeding difficulties in DS, other factors are also likely to contribute.

In some children with DS, feeding difficulties and gastrostomy insertion followed an episode of status epilepticus. It is well recognized that some children with DS experience long-term neurologic sequelae following status epilepticus^24^ of which feeding difficulties may form a part. Thus, although feeding difficulties in DS can emerge throughout life, potential contributing factors may change with age, such as the influence of status epilepticus, which is more common in childhood in DS.^25^

The identification of risk factors for the development of feeding difficulties was limited in this study by the retrospective design and methods of data collection. Medical records can lack detail and temporal accuracy, and feeding difficulties may not be recorded during appointments, most of which were for the individuals' epilepsy. In the adult DS cohort only, 3 individuals were recorded to have developed feeding difficulties during childhood. This is incongruous with findings from the pediatric DS cohort, and the DSUK survey cohort, where the onset of feeding difficulties was commonly in childhood. This likely in part reflects reporting bias, as well as the limitations of using retrospective analysis of electronic medical records to obtain early medical and childhood history in adults affected by chronic, childhood-onset conditions such as DS, where the comprehensive pediatric history is frequently not available.^26^ It is possible that some individuals in the adult DS cohort developed feeding difficulties during childhood, but that information was not recorded in their available electronic medical records.

It is probable that various clinical and treatment-related factors, many of which we were unable to explore in this retrospective, observational study, contribute to the development of feeding difficulties and the need for gastrostomy in DS. A potential contribution of seizure frequency and/or severity, and history of status epilepticus to the development of feeding difficulties were not explored because of the challenges of using a retrospective study to obtain accurate contemporaneous information about seizures in relation to the development of feeding difficulties. It is possible that the associations identified in this study between treatment with various ASMs and the development of feeding difficulties instead reflect an underlying clinical variable such as seizure frequency and/or severity which was not explored. Further prospective work is required to explore factors associated with the development of feeding difficulties in DS.^15^

Almost all individuals in the survey cohort reported feeding difficulties, and almost half already had a gastrostomy, likely reflecting ascertainment bias. Caregivers were asked to recall information from the time of gastrostomy insertion (a median of 6 years before survey completion), and therefore, there may be inaccuracies.

We acknowledge that this is a UK-based study, and although feeding difficulties are reported in DS caregiver surveys across multiple countries (eTable 1), international variation in the effect of feeding difficulties in DS is observed,^27,28^ which may reflect both international differences in DS management practices, and cultural differences surrounding feeding. In addition, individuals in the combined DS cohort were identified from 2 tertiary epilepsy centers and therefore may represent individuals at the more severely affected end of the DS spectrum.

Feeding difficulties in DS can have considerable consequences, prompting investigations or hospital admission. Weight loss/failure to gain weight was reported in more than a third of individuals and was the second most common reason for gastrostomy. Problems with growth and weight have previously been reported by DS caregivers in up to 57% of individuals,^4,5^ and children with DS have been shown to have shorter stature, and lower body weight, compared with matched population norms,^29^ the reasons for which have not been established. In the adult DS cohort, marked weight loss and low BMIs were recorded, with many meeting criteria for the diagnosis of malnutrition.^30^ For those in whom investigations were undertaken, no cause for the often dramatic weight loss was identified. Individuals with neurodevelopmental disorders are at increased risk of malnutrition,^31,32^ which may go underrecognized, particularly in adults in residential care.^33^ Malnutrition has widespread adverse effects, is associated with increased rates of morbidity and mortality in hospital patients, and can lead to substantial increases in healthcare costs.^34^ In the United Kingdom, the Confidential Inquiry into Premature Deaths of People with Learning Disabilities highlighted that a greater than expected number of individuals with intellectual disability were either underweight or obese, and inadequate knowledge about nutrition by care staff led to a lack of recognition of malnutrition in some deaths.^35^ Twelve percentage of people whose deaths were reviewed had a gastrostomy; in these individuals, there were serious concerns around the length of time between the decision that a gastrostomy was needed and the actual procedure taking place.^35^

The average time between feeding symptom onset and gastrostomy insertion in this study was 1–1.5 years, but some individuals experienced feeding difficulties for several years before the procedure. Details regarding the severity of feeding difficulties, and the timing of clinical decision-making around gastrostomy, were not available for most, and therefore, we are unable to comment on whether delays were unacceptable and what factors might have contributed. Decision-making surrounding gastrostomy can be challenging and may be protracted as concerns relating to the procedure, and beliefs around the meaning and importance of feeding by mouth are addressed.^36^ This may potentially lead to delays in intervention,^15^ increasing the risks associated with malnutrition, dehydration, aspiration, and/or suboptimal medication compliance. We show that there was a high level of concern experienced by DS caregivers before gastrostomy, encompassing a wide range of issues regarding the procedure and life with a gastrostomy. Caregivers reported that access to information was lacking, and the need for information specific to gastrostomy in people with DS was emphasized. Negative caregiver experiences were related to a lack of awareness, understanding, and support from healthcare providers about the feeding difficulties being experienced by their child (eTable 7). There is a need for caregivers to be provided with information about feeding difficulties and the potential need for gastrostomy in DS early on to help mitigate anxiety surrounding challenging feeding^15,37^ to facilitate shared decision-making about gastrostomy^36,37^ and to prevent unnecessary delays in intervention.

Gastrostomy-related complications were frequently observed in individuals with DS but occurred at rates in keeping with those reported in other conditions (17–38% for periprocedural^38,39^ and 26–67% for long-term complications^38-41^). The majority of complications were considered minor,^42^ with most being avoidable with high-quality gastrostomy aftercare.^43^ Systemic infections are considered major complications following gastrostomy insertion.^42^ The 4 individuals reporting periprocedural infections in this study may have developed systemic infections, giving a major complication rate of 9% (across all cohorts), which is comparable with published rates in other conditions (2–13%^38,44^). Systemic infections (particularly pneumonia) postgastrostomy may be more common in individuals with neurologic disease,^45^ meaning that those with DS may be at greater risk.

Despite a wide range of concerns expressed by DS caregivers before gastrostomy, and a high frequency of reported minor complications, caregiver opinions and experiences of gastrostomy were compellingly positive, and overall outcomes were favorable, with improvements in medication compliance, weight, seizure control, cognition, overall health, quality of life, and a reduction in hospital admissions. This is in keeping with findings in children with drug-resistant epilepsy, where gastrostomy placement, primarily to facilitate ASM administration, led to improved seizure control and quality of life.^14^

Among the DEEs, feeding difficulties and the need for gastrostomy are not unique to DS.^9,46^ Multiple clinical, demographic, and treatment-related factors, some shared between neurodevelopment disorders, are likely to contribute to feeding difficulties in these conditions.^15,46^ Syndrome-specific factors may also be present, including potential contribution from the underlying disease pathophysiology.^15,46^ Further work is required to fully elucidate the underlying mechanisms and risk factors for feeding difficulties in DS and other DEEs.

### Summary and Recommendations

In this retrospective, observational study, we show that feeding difficulties are common in DS and gastrostomy is frequently required. Caregivers should be provided with information about feeding difficulties and the potential need for gastrostomy early on to help mitigate anxiety and to avoid unnecessary delays in intervention when required. During a clinical review of people with DS, feeding difficulties should be proactively sought and weight, BMI, and/or growth charts should be monitored. Reversible contributing factors should be addressed, and multidisciplinary input with SALT, dieticians, and other appropriate specialists should be arranged promptly. Although a wide range of feeding symptoms were reported in those with, and without, gastrostomy in this study, individuals demonstrating medication refusal (typically alongside poor appetite) were 6 times more likely to require a gastrostomy, and therefore, it is important to be particularly vigilant for this combination of symptoms. Gastrostomy can be used to safely and effectively manage feeding difficulties in people with DS, resulting in many positive outcomes, and should be considered where necessary.

## Appendix Authors

**Table:**

Name	Location	Contribution
Lisa M. Clayton, MRCP, PhD	UCL Queen Square Institute of Neurology, London; Chalfont Centre for Epilepsy, Bucks, United Kingdom	Drafting/revision of the manuscript for content, including medical writing for content; major role in the acquisition of data; study concept or design; analysis or interpretation of data
Bahar Azadi, MBBS, PhD	Great Ormond Street Hospital, London, United Kingdom	Drafting/revision of the manuscript for content, including medical writing for content; major role in the acquisition of data
Claire Eldred, MA	Dravet Syndrome UK, Registered Charity Number 1128289, Member of Dravet Syndrome European Federation, Chesterfield, United Kingdom	Drafting/revision of the manuscript for content, including medical writing for content; major role in the acquisition of data; study concept or design
Galia Wilson	Dravet Syndrome UK, Registered Charity Number 1128289, Member of Dravet Syndrome European Federation, Chesterfield, United Kingdom	Drafting/revision of the manuscript for content, including medical writing for content; major role in the acquisition of data; study concept or design
Robert Robinson, MRCPCH, PhD	Great Ormond Street Hospital, London, United Kingdom	Drafting/revision of the manuscript for content, including medical writing for content
Sanjay M. Sisodiya, FRCP, PhD	UCL Queen Square Institute of Neurology, London; Chalfont Centre for Epilepsy, Bucks, United Kingdom	Drafting/revision of the manuscript for content, including medical writing for content; study concept or design
